# Recent Advances in Synthetic Methodologies to Form C-^18^F Bonds

**DOI:** 10.3389/fchem.2022.883866

**Published:** 2022-04-14

**Authors:** Zhiyi Liu, Yijun Sun, Tianfei Liu

**Affiliations:** ^1^ State Key Laboratory of Elemento-Organic Chemistry, Department of Chemistry, Nankai University, Tianjin, China; ^2^ The Haihe Laboratory of Sustainable Chemical Transformations, Nankai University, Tianjin, China; ^3^ Key Laboratory of Organofluorine Chemistry, Shanghai Institute of Organic Chemistry, Chinese Academy of Sciences, Shanghai, China

**Keywords:** radiofluorination, organic synthetic methodologies, leaving functional groups, transition metal catalysis, photocatalysis, electrocatalysis

## Abstract

Positron emission tomography (PET) is an important technique for the early diagnosis of disease. Due to the specific physical and chemical properties of Fluorine-18, this important isotope is widely used in PET for labelling and molecular imaging, and its introduction into medicine molecules could produce PET tracers. Developing with the development of organic synthetic methodologies, the introduction of Fluorine-18 into drug molecules efficiently and rapidly under mild conditions, and the formation of C-^18^F chemical bonds, has become one of the leading topics in both organic synthetic chemistry and radiochemistry. In this mini-review, we review a series of recent advances in the organic synthesis of C-^18^F bonds (2015–2021), including non-catalytic radiofluorinations via good leaving functional groups, transition metal-catalyzed radiofluorinations, and photo- or electro-catalytic synthetic radiofluorinations. As a result of the remarkable advancements in this field, organic synthetic methods for forming C-^18^F bonds are expected to continue growing.

## Introduction

Positron emission tomography (PET) is a non-invasive technology for radionuclide imaging, and it is sensitive to and informative of biological processes *in vivo*. A significant advantage of PET technology is that it is non-invasive, and that this powerful tool has been used not only for the diagnosis of various cancers (Phelps, 2000) but also the study of plant science (McKay et al., 1988; Kang et al., 2009; Niu et al., 2020), bacterial infections (Northrup et al., 2019; Welling et al., 2019) and even the subsurface microbial processes of soils (Kinsella et al., 2012).

**FIGURE 1:**
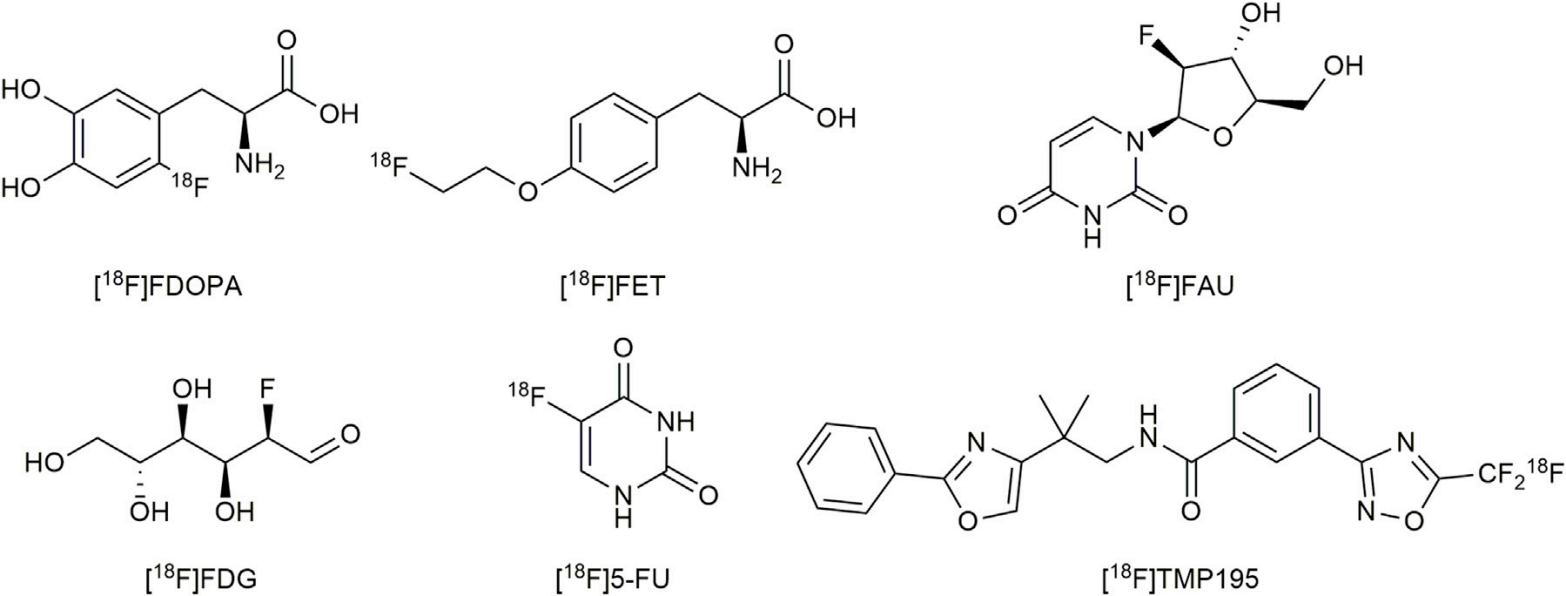
Some examples of [^18^F]-labelled PET tracers. RCC, radiochemical conversion; EDG, electron-donating group; DG, directing group.

PET applications in the previously mentioned areas require appropriate positron-emitting radioisotopes and appropriate compounds labeled with the radioisotopes. Internally used positron-emitting radioisotopes should have a long half-life (t_1/2_), low-energy positron emission, and flexible chemistry properties. Fluorine-18 [^18^F] is a positron-emitting radioisotope of fluorine, considered as an important source of positrons. The mass of ^18^F is 18.0009380 (6) u and its half-life is 109.771 (20) minutes. Furthermore, this radioisotope has low positron energy (0.64 MeV) and high efficiency by positron emission (97%) (Qaim, 2017). In medicinal chemistry, fluorine atoms are considered as bioisosteres of hydrogen atoms (Patani and LaVoie, 1996). In summary, all specific properties of Fluorine-18 previously mentioned fit perfectly for the requirements of its applications in PET (Qaim, 2017). Various [^18^F]-labelled compounds are investigated as PET tracers, including amino acids and their derivatives, peptides, saccharides, small medicinal molecules, and so on (shown in Figure 1). Introducing Fluorine-18 atoms into these molecules remains intractable and challenging, especially molecules with complex structures and various functional groups. Therefore, effective and convenient radiofluorination reactions for the synthesis of PET tracers are required. The reactions should be performed at a late stage of synthesis to reduce the unproductive radioactive decay before injections with radiochemical yields (RCYs) > 5% to support meaningful PET imaging (Lee et al., 2011). The radiotracers should be obtained in sterile solutions suitable for injection. [^18^F] is generated by a cyclotron in the form of [^18^F]fluorine gas ([^18^F]F_2_) via ^18^О(p,n)^18^F or ^20^Ne (d,α)^18^F reactions, or obtained from proton bombardment of ^18^O–enriched water in the form of [^18^F]fluoride via ^18^О(p,n)^18^F reactions. Recent research mainly focused on using [^18^F]fluorides as fluorine donors, which is more convenient to handle than extremely dangerous [^18^F]F_2_. Because the C-F bonds are restricted to enzyme catalysis *in vivo*, the formation of the C-F bond has been widely studied as a strategy for radiofluorination of PET tracers (Preshlock et al., 2016).

In this mini-review, we summarize recent advances from 2015 to 2021 in synthetic methodologies to form C-[^18^F] bonds. The following sections will include three main topics: 1) radiofluorination of substrates with good leaving groups, 2) transition metals catalyzed radiofluorination, 3) Photo- and electro-catalytic methods for radiofluorination. Hopefully, this mini-review will shed light on new methods for developing radiofluorination methods in the future.

## Non-Catalytic Radiofluorinations of Substrates with Good Leaving Groups

### Non-catalytic Radiofluorinations Applying Direct Aryl C-F Bond Formation

Although fluorination via the S_N_Ar mechanism to form C-F bonds appears to be the easiest fluorination method in the absence of catalysts, Balz-Schiemann reactions (Nozaki and Tanaka, 1967) and Wallach reactions (Kovac et al., 2013) demonstrated low radiofluorination conversion factors (RCYs). Additionally, the requirement for the aromatic precursors in these reactions to have an electron-withdrawing group in the ortho or para position to the leaving group restricts the substrate scope, which means that the development of new precursors is a priority for non-catalytic radiofluorination. It was reported in 2012 that a new radiofluorination strategy to form C-F bonds using diphenyl group instead of dimethyl group (Maeda et al., 1987) was developed, which expanded the substrates scope and maintained high RCYs (Table 1, #1) (Mu et al., 2012). Applying the same strategy, Årstad et al. developed a practical method for aromatic radiofluorination of drug-like molecules, which showed high labelling efficiency (Table 1, #2) (Sander et al., 2015). Consequently, in 2018, they reported dibenzothiophene sulfonium salt as leaving groups under mild conditions (Table 1, #3) (Gendron et al., 2018). Besides sulfonium salts, Pike and colleagues discovered diaryl sulfoxides with electron-withdrawing groups could also serve as precursors and yield high RCYs (Table 1, #4) (Chun et al., 2013).

**TABLE 1:** Non-catalytic labelling reactions of substrates with good leaving groups.

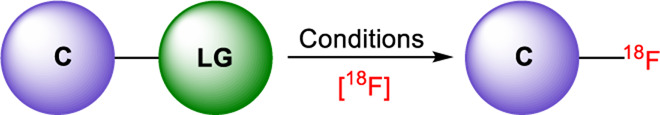						
Entry	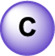 LG(Leaving Groups)		Anions	Conditions	Yields	References
1	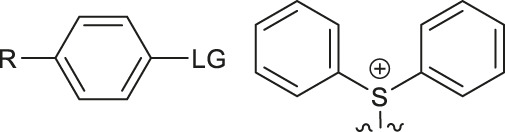		TfO^−^	[^18^F]KF/K_222_	7 examples RCCs = 0–99%	Mu et al. (2012)
				K_2_CO_3_ or Cs_2_CO_3_		
				DMF,90–110°C, 15 min		
2	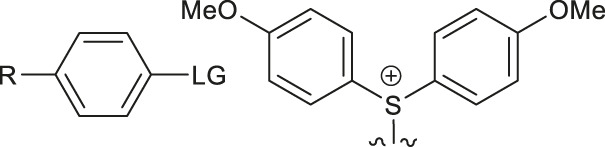		TfO^−^	[^18^F]KF/K_222_	11 examples RCYs = 0–88%	Sander et al. (2015)
				KHCO_3_, DMSO, 110°C, 15 min		
3	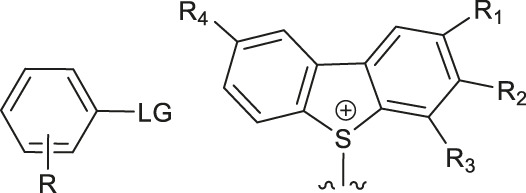		TfO^−^	[^18^F]Fluoride, K_222_/KHCO_3_,DMSO, 110°C, 15 min	19 examples RCYs = 1.7–89%	Gendron et al. (2018)
4	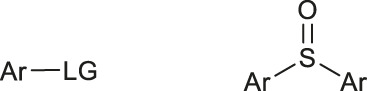		—	^18^F^-^, K_2_CO_3_/K_222_ DMF,200°C	20 examples RCCs = 0–98%	Chun et al. (2013)
5	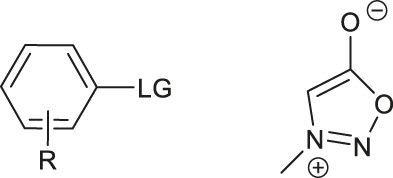		—	[^18^F]Fluoride, Et_4_NHCO_3_,DMSO, 150°C, 5 min	18 examples RCYs = 21–98%	Narayanam et al. (2017)
6	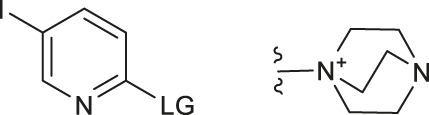		TfO^−^	(1) [^18^F]F^-^MeOH	RCY=77 ± 7%	Humpert et al. (2021)
				(2) evaporatrion of MeOH 80°C, 10 min		
				(3)DMSO, 100°C, 15 min		
				(4)RP SPE		
7	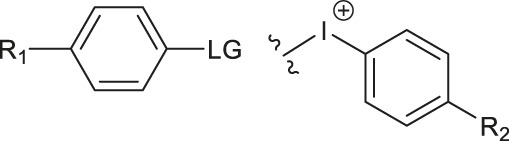		Cl^−^, Br^−^,TfO^−^	[^18^F]KF/K_222_ CH_3_CN,85-110°C 35–40 min	12 examples RCYs = 7.5–88%	Pike and Aigbirhio, (1995)
			CF_3_CO_2_			
8	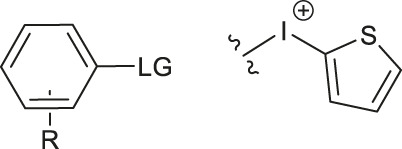		Br^−^, I^−^	[^18^F]KF/K_222_ DMF, 130°C	9 examples RCYs = 17–75%	Ross et al. (2007)
			TsO^−^, TfO^−^			
9	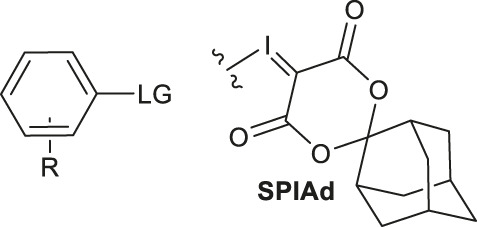		—	[^18^F]Fluoride, TEAB DMF, 120°C, 10–20 min	54 examples RCYs = 33–56%	Rotstein et al. (2014); Liang et al. (2019)
10	ArH_2_C-LG	Cl,-Br, I	—	PDFA, S_8_[^18^F]KF/K_222_ DMF, 70°C, 1 min	4 examples RCCs = 33–56%	Zheng et al. (2015)
11	Ars-LG	Ar-s	—	CHF_2_^18^FtBuOK, DMF 20°C	11 examples RCYs = 45–75%	Carbonnel et al. (2017)
	Arse-LG	Ar-se				
12	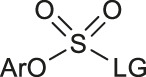		—	^18^F^−^, K_222_ K_2_CO_3_, CH_3_CN,23°C 30 s	25 examples RCYs = 83–100%	Zheng et al. (2021)

Notes: a) LGs are substituted by -^18^F, except b. b) LGs are substituted by –[^18^F]SCF_3_.

The radiofluorination reactions to other leaving groups have also been investigated. N-arylsydnones are important precursors that provide excellent RCYs, in which sydnone is an excellent activated group that could not stabilize an incoming negative charge through resonance delocalization. This was reported by Murphy’s group in 2017 (Table 1, #5) (Narayanam et al., 2017). DABCO was studied as another novel leaving group by Bernd Neumaier’s group in 2021 (Humpert et al., 2021). 5-iodo-2-[^18^F]fluoropyridine can be synthesized from 2-DABCO-5-iodopyridine precursor. This precursor can be prepared from commercially available 5-iodo-2-hydroxypyridine, according to the procedure reported by (Richard et al., 2020). The [^18^F]-labelled molecules were isolated by reversed-phase solid-phase extraction (RP SPE) strategy, with high RCYs of 77 ± 7% (n > 10) and radiochemical purity (>99%), and was further conjugated with thiol, using XantPhos Pd G3 as a catalyst with the RCYs varying from 10% to >90% (Table 1, #6). The author successfully applied the Pd-catalyzed S-arylation protocol to several short peptides under mild aqueous conditions with rapid kinetics. However, its unstable RCYs depending on the structures of peptides need to be overcome.

Meanwhile, diaryliodonium salts are a range of essential precursors in radiofluorinations reactions. In 1995, Pike and Aighirhio reported the first example of diaryliodonium salts precursors giving high RCYs, revealing that electron-deficient arenes were easier to be labelled (Table 1, #7) (Pike and Aigbirhio, 1995). In 2007, Coenen et al. developed a new precursor, aryl (2-thienyl) iodonium salts, and found this method could introduce [^18^F] to electron-rich arenes with high regiospecificity (Table 1, #8) (Ross et al., 2007). In 2010, Lee, Pike, and co-workers found that the reactions of [^18^F]fluoride ions with unsymmetrical diaryliodonium salts complied with the Curtin-Hammett principle (Chun et al., 2010). This mechanism was proved by crystal structures of diaryliodonium fluorides in 2017 (Lee et al., 2017).

Aryl iodonium ylides can also be used as precursors of radiofluorination reactions. In 2014, Ermert et al. applied aryl iodonium ylides with Meldrum’s acid auxiliaries in the radiofluorination of arenes (Cardinale et al., 2014). In the same year, Liang et al. designed spirocyclic iodonium ylides as precursors, and they proved that spirocyclic iodonium ylides were able to radiofluorinate non-activated and sterically hindered arenes and tolerate a range of functional groups with high RCYs and regioselectivity (Table 1, #9) (Rotstein et al., 2014; Liang et al., 2019). The same group found a better auxiliary **SPIAd** with superior precursor stability under radiofluorination conditions, and confirmed that iodonium (III) ylides performed better than diaryliodonium (III) salts by DFT calculations in 2016 (Rotstein et al., 2016). And in 2017, Riss et al. suggested the addition of PPh_3_ and N_2_ protection could further increase fluorination yields and reaction rates of the method (Jakobsson et al., 2017).

### Non-catalytic Radiofluorinations to Form Other [^18^F]-Labelled Functional Groups

Since the -SCF_3_ functional group has a very high lipophilicity (*π* = 1.44), the introduction of Ar-SCF_2_^18^F structure into medicinal molecules can dramatically affect their cell-membrane permeability (Xu et al., 2015). Therefore, the radiofluorination of this structure has been a research focus. Zheng et al. (2015) reported a new method to introduce the ^18^F-trifluoromethylthiol group in the presence of [^18^F]-fluoride, S_8_, and difluoromethylene phosphobetaine (PDFA), which resulted in formation of the ^18^F-labeled SCF_3_ anion *in situ* from difluorocarbene (Table 1, #10). (Carbonnel et al., 2017) used [^18^F]-fluoroform and disulfides to prepare ^18^F-labelled aryl-SCF_3_ with high RCYs, and they found that this method could be used for diphenyl diselenides (Table 1, #11).

Recent research has shown that sulfuryl fluorides can also be used for radiofluorination. In 2021, Zheng et al. (2021) reported the synthesis of aryl [^18^F]fluorosulfates via sulfur fluoride exchange (SuFEx) click reactions (Table 1, #12). This method show excellent RCYs and high molar activity under mild conditions only within 30 s.

## Transition Metals Catalyzed Radiofluorinations

### Copper-Mediated/Catalyzed Radiofluorinations

**FIGURE 2:**
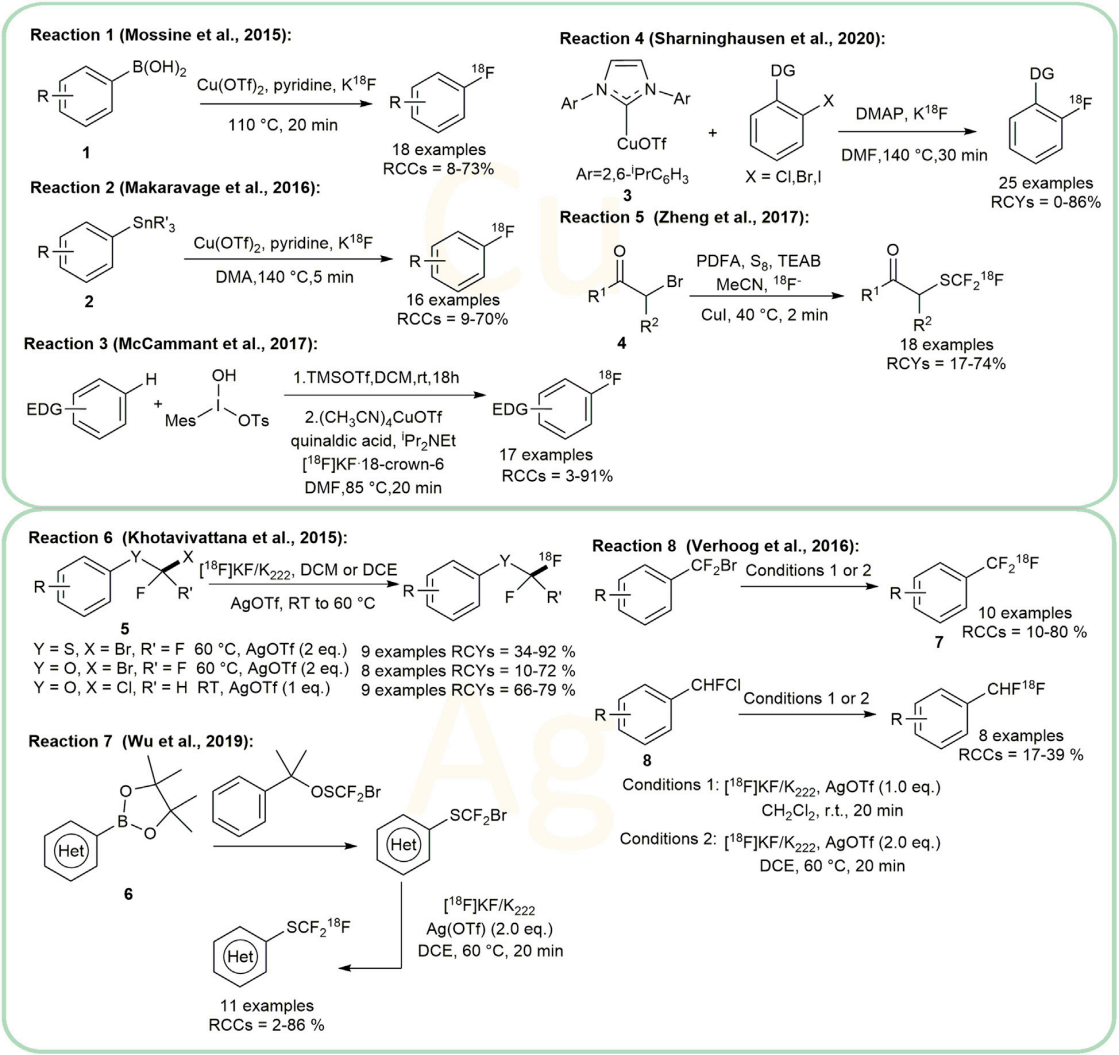
Cu and Ag mediated/catalyzed radiofluorination.

Some different approaches based on the direct formation of the C-CF_2_^18^F bond of arenes and heteroarenes under copper-mediated/catalyzed conditions have been reported by Gouverneur, Riss, Vugts, Jubault and Labar before 2015 (Huiban et al., 2013; Ivashkin et al., 2014; Ruhl et al., 2014). Copper catalysts have been widely used to mediate or catalyze aromatic fluorination reactions with aryl boronates, aryl boronic acids, (mesityl)-(aryl)iodonium salts, and arylstannanes since 2014 (Tredwell et al., 2014; Guibbal et al., 2020; Mossine et al., 2020). In 2015, Sanford and Scott’s group developed an inexpensive copper salt, Copper (II) trifluoromethanesulfonate (Cu(OTf)_2_), providing 8–73% RCCs in the radiofluorination of aryl, heteroaryl, and vinyl boronic acid precursors **1** for the first time (Mossine et al., 2015). This method presented good functional group tolerance and water tolerance (Figure 2, Reaction 1). Still utilizing Cu(OTf)_2_ as catalyst, Makaravage et al. (2016) developed a copper-mediated ^18^F- fluorination method of arylstannanes **2** with high specific activity. This method was the first practical nucleophilic ^18^F-fluorination to stannanes and was compatible with both electron-rich and electron-deficient arene substrates (Figure 2, Reaction 2). Notably, as the residual metal levels were below the allowed limits set by ICH guidelines and the protocol was readily automated, it showed a great potential in medicinal PET applications.

Mesityl-aryl iodonium salt was also applied as the precursors in the copper-mediated ^18^F- fluorination to produce ^18^F-arenes under mild conditions before 2015 (Ichiishi et al., 2014). In 2017, Sanford and co-workers took their work further (McCammant et al., 2017). Generated *in situ* from stable and easily available C−H starting materials, the unstable electron-rich diaryliodonium salts intermediates were obtained and subsequently converted to Ar-^18^F to perform C-H bond activation (Figure 2, Reaction 3). This method tolerates a wide range of electron-rich arenes. Yet, protic functional groups were not compatible and thus pre-protection is required. Notably, the lower selectivity of the method to heterocycles would probably limit its applications.

In 2020, the same group disclosed a new ^18^F- fluorination method of aryl halides with directing groups (DG) at the ortho position (Sharninghausen et al., 2020). The reaction utilized copper N-heterocyclic carbene complexes **3** as mediators. The N-heterocyclic carbene stabilized Cu(I)− ^18^F complexes, which could accelerate the key step of aryl-bromide bond activation (Figure 2, Reaction 4).

In 2016, by introducing copper catalyst, Zheng et al. (2017) found that ɑ-bromocarbonyl compounds **4** could also be converted to ɑ-[^18^F]SCF_3_ carbonyl derivatives (Figure 2, Reaction 5). This strategy broadened the substrate scope of their former investigations (Table 1, #10).

### Ag Mediated/Catalyzed Radiofluorination

A series of radiofluorination reactions mediated by Ag had already been reported by Gouverneur’s group before 2015 (Teare et al., 2010; Stenhagen et al., 2013). In 2015, they used AgOTf and [^18^F]KF/K_222_ to radiofluorinate aryl -OCF_3_, -SCF_3_, and -OCHF_2_ structures **5** at room temperature to 60°C in 20 min (Figure 2, Reaction 6) (Khotavivattana et al., 2015). Additionally, the protocol accepts aryl cores containing alkyl, ethers, esters, aryl, halogens, and unprotected amines. According to the author, the order of reactivity towards ^18^F-fluoride is: ArOCHFCl > ArCF_2_Br ≈ ArCHFCl > ArSCF_2_Br > ArOCF_2_Br. Later, in 2019 Shen’s and Gouverneur’s groups extended the protocol to the synthesis of heteroarenes started from commercially available aryl-Bpin (boronic acid pinacol ester) substrates **6** (Figure 2, Reaction 7) (Wu et al., 2019).

Gouverneur’s group synthesized ^18^F-labeled aryl-CF_3_ and aryl-CHF_2_ structures from aryl-CF_2_CO_2_H and aryl-CHFCO_2_H with AgNO_3_ in 2013 (Mizuta et al., 2013). They described a protocol for obtaining ^18^F-labeled aryl-CF_3_ products **7** using AgOTf and [^18^F]KF/K_222_ in 2016 (Verhoog et al., 2016), in which AgOTf improved the RCCs of various substrates tremendously. This method has been extended to aryl-CHF_2_, for which no [^18^F] labelling methods are available. They succeeded in applying aryl-CHFCl **8** as precursors (Figure 2, Reaction 8) under mild conditions.

### Ni Catalyzed Radiofluorination

**FIGURE 3:**
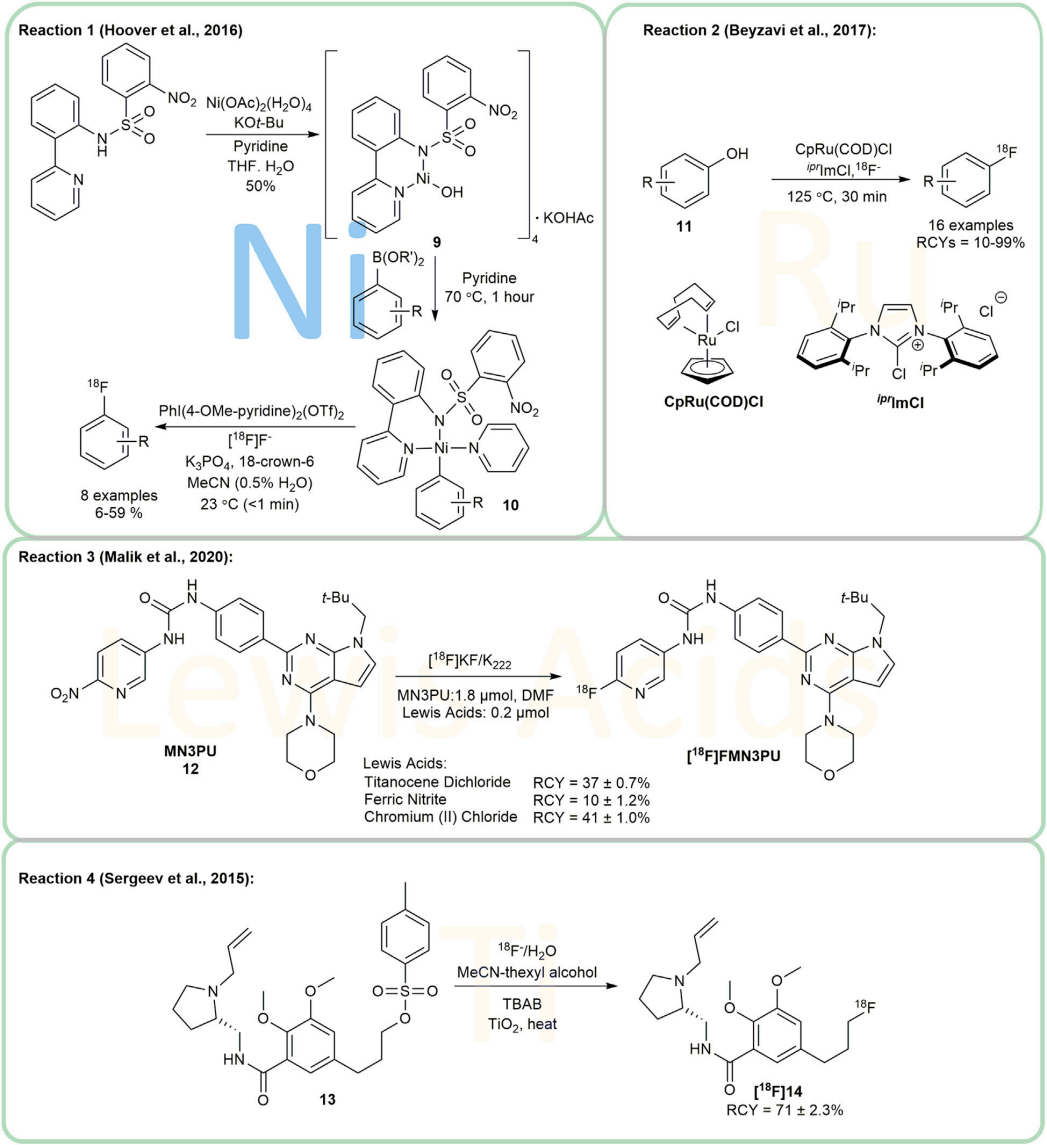
Ni, Ru and other transition metal mediated/catalyzed radiofluorination.

A nickel-mediated protocol for aryl-[^18^F] labeling was presented by Hooker’s and Ritter’s group in 2012 (Lee et al., 2012). They developed and applied their nickel-mediated protocol in 2016, compound **9** being utilized to produce [Ni]-Ar **10** in pyridine from aryl boronic acids and esters (Figure 3, Reaction 1). This method was successfully applied to the synthesis of [^18^F]5-fluorouracil, a PET tracer for clinical research in oncology for the first time. However, the isolated yield of the entire synthetic route needs to be improved (Hoover et al., 2016).

### Ru Catalyzed Radiofluorination

Ritter’s group investigated ruthenium complexes as transition metal catalysts for the radiofluorination of phenols **11** for the first time in 2017 (Figure 3, Reaction 2) (Beyzavi et al., 2017). Their work enriched the tools for ^18^F-labeling of electron-rich aryl systems. The method is suitable for heterocyclic compounds as well as a wide variety of functional groups. According to the report, several basic amines performed well, and the reaction show good tolerance to moisture and air. The authors suggested, however, that heterocycles might interfere with the formation of efficient ruthenium complexes, which might be improved through optimization of ligand structures.

### Other Transition Metal-Catalyzed Radiofluorination

**MN3PU 12**, which targets leucine repeat kinase 2 (LRRK2), is of great significance in the investigation of Parkinson’s disease. However, **12** is temperature-sensitive and thermally decomposes in polar, aprotic solvents (e.g., DMF and DMSO), thus causing undesirable byproducts under the general conditions of radiofluorination. Schaffer et al. (Malik et al., 2020) demonstrated that by using chromium (II) chloride and titanocene dichloride as Lewis acids, it was possible to achieve non-decay corrected radiochemical yields (ndc RCYs) for [^18^F]MN3PU up to 41 ± 1% and 37 ± 0.7%, respectively (Figure 3, Reaction 3).

Using TiO_2_ nanoparticles as the catalyst (Sergeev et al., 2015) radiofluorinated aromatic, aliphatic, and cycloaliphatic substrates with a maximum RCC in the presence of up to 25% water (Figure 3, Reaction 4). The tosyl-fallypride **13** was selected as the model compound and was reacted in a mixture of acetonitrile and thexyl alcohol at 110°C for 7 min to yield [^18^F]fallypride **[** ^**18**^ **F]14**, a highly specific radio-probe used in PET imaging of the brain. Interestingly, the authors noticed that some [^18^F]fluoride was trapped on the catalyst and could not be removed. Furthermore, they determined the optimal reaction conditions and investigated a range of substrates with high RCCs above 70%. Additionally, the group performed full production runs in order to illustrate the overall RCY of isolated [^18^F]Fallypride as an injectable product. Despite the hypothesised mechanism, more research on its mechanism and substrate scope is required.

### Photo- and Electro-Catalytic Methods for Radiofluorination

**FIGURE 4:**
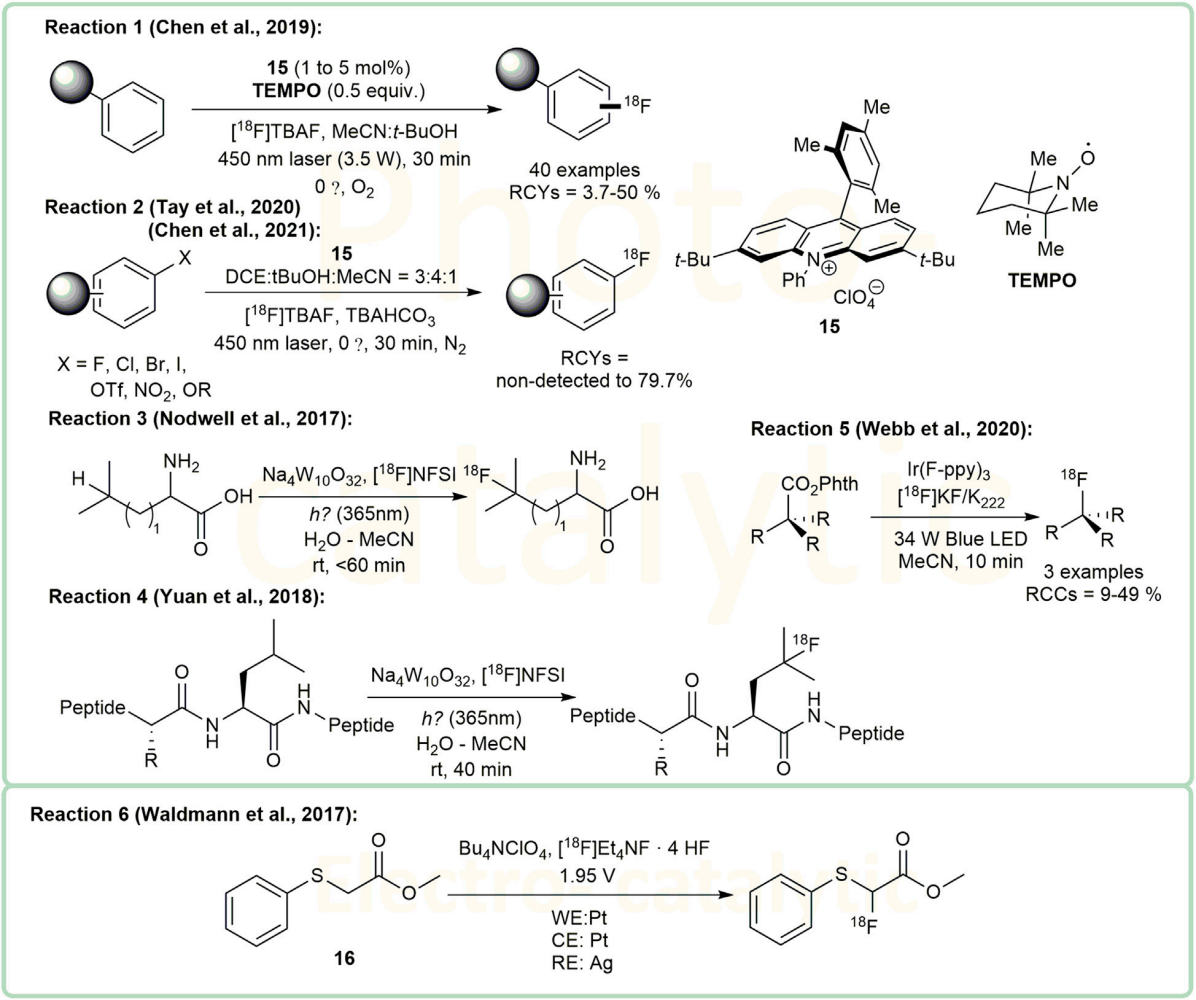
Examples of photo- and electro-catalytic methods for radiofluorination.

The introduction of 18F into organic compounds via Photo- and Electro-catalytic methods under mind conditions is a very attractive topic (Bui and Kim, 2021; Hernández-Valdés and Sadeghi, 2021). Using cationic organic dye **15** as a photoredox catalyst, Nicewicz and colleagues developed a series of photoredox catalytic methods to form C-^18^F bonds. In 2019, they reported a direct arene C-H radiofluorination catalyzed by this cationic organic dye system under 450 nm laser (Chen et al., 2019) (Figure 4, Reaction 1). According to their work, due to steric effect, ^18^F-labeling of methoxy aryl substituents at the para position is preferred unless the para position is occupied by another functional group. In addition to direct arene C-H radiofluorination, nucleophilic aromatic substitution was investigated subsequently. They have also applied this catalyst to the aromatic halides and their derivatives, with leaving groups such as F, Cl, Br, I, OTf, NO_2_ (Chen et al., 2021) and OR (R = *para*-chlorophenyl) (Tay et al., 2020) (Figure 4, Reaction 2). This method did not perform well when X was the iodine substituent, and the RCYs of reactions varied from non-detected to 79.7%. For the radiofluorination on aliphatic derivatives, Sodium decatungstate (Na_4_W_10_O_32_) has been investigated as a photoredox catalyst. Britton and colleagues demonstrated that this catalyst can radiofluorinate the unprotected branched aliphatic amino acids at the branched positions under mild conditions. In their report, amino acids including leucine, homoleucine and *β*-amino-homoleucine displayed RCYs of 23.3 ± 3.3%, 27.9 ± 3.3% and 29.8 ± 0.7% repectively, while valine and isoleucine showed unsatisfactory RCYs (<6.4%) (Nodwell et al., 2017) (Figure 4, Reaction 3). Later, considering that peptides are often ideal ligands for diagnostic molecular imaging, the same group also utilized this method to synthesize the ^18^F-labelled peptides containing leucine residues, including [^18^F]FAfLGEA-NH_2_ (a ligand for cancer-specific receptor EGFRvIII) [^18^F]ZJ-43 (a NAAG peptidase inhibitor) and the analogue of [^18^F]ZJ-43 (Yuan et al., 2018) (Figure 4, Reaction 4). Their results indicated that their method had great potential in the synthesis of PET tracers and chemical biology. In 2020, Doyle and coworkers reported a photocatalytic nucleophilic radiofluorination of redox-active esters in presence of Ir(F-ppy)_3_ under the irradiation of a 34W blue LED (Webb et al., 2020) (Figure 4, Reaction 5). Their protocol provided a new strategy for the radiofluorination of bioactive molecules.

Research on ^18^F-labeling by electrosynthesis provides a new direction for investigations into radiofluorination (Waldmann et al., 2017; Hernández-Valdés and Sadeghi, 2021). Professor Sadeghi’s group studied the electrochemical fluorination of di-*tert*-butyl-(4-*tert*-butyl-1,2-phenylene)-dicarbonate (He et al., 2015) and methyl (phenylthio)acetate (Balandeh et al., 2017; Balandeh et al., 2018). These studies led to the invention of an automated apparatus for ^18^F-fluorination of organic molecules **16** (Figure 4, Reaction 6) by his group (Waldmann et al., 2017), reported to achieve the highest RCY of 17.9%. According to the report, the reaction voltage was set to 1.95V vs. a silver pseudo-reference electrode. One of the highlights of these reactions were their high radiochemical purities (>99%). However, the drawback of the apparatus is the high proportion of the starting activity that is unreacted (48%) and remaining in the system (38%), which needs further improvement.

## Concluding Remarks and Outlook

Introducing C-^18^F bonds into organic compounds with high efficiency, rapidity, and simplicity under mild conditions has always been a topic of great interest in medicinal chemistry and organic synthesis chemistry, and this topic still presents various opportunities and challenges. When forming C-^18^F bonds for PET tracers, radiochemists must take functional group tolerance into account. The above summary shows that non-catalytic radiofluorination, transition metal catalysts, photo- and electrocatalytic strategies, and using good leaving functional groups have all been well developed in the last 5 years. In the meantime, late-stage radio-modification of drug-like molecules and PET tracers is still a great prospect, especially rapid and highly selective direct radiofluorination. Throughout the next five to 10 years, it will be exciting to see how this field develops to achieve direct and late-stage radiofluorination of drug-like molecules and PET tracking molecules with C-^18^F bonds.
